# Outcome of Combined Neoadjuvant Chemotherapy and Vitamin A in Advanced Cervical Carcinoma: A Randomized Double-Blind Clinical Trial

**DOI:** 10.31557/APJCP.2019.20.7.2213

**Published:** 2019

**Authors:** Rizal Sanif Sanusi

**Affiliations:** *Division of Gynecologic Oncology, Department of Obstetrics and Gynecology, General Hospital of Dr. Mohammad Hoessin, University of Sriwijaya, Palembang, Sumatra, Indonesia. *

**Keywords:** Cervical cancer- NAC- neoadjuvant chemotherapy- nutrition- vitamin A

## Abstract

**Background:**

The latest World Health Organization (WHO) inquiry on the epidemiology of cervical cancers indicate there are approximately 528,000 new cases per year, ranking fourth after breast, colorectal and lung cancer. The validity of neoadjuvant chemotherapy (NAC) alone in advanced cervical cancer is still being debated. NAC induces tumor shrinkage prior to pursuing surgery. NAC also has the benefit of sterilizing the lymph nodes and parametria, thereby lowering the need for adjuvant therapy after surgery. This research aims to determine the impact on the treatment of advanced cervical carcinoma with NAC, with the additional provision of Vitamin A during treatment to assess the factors that could affect the outcome of clinical treatment.

**Methodology:**

The research methodology and design of this study is a randomized double-blind clinical trial to compare the effects of treatment with NAC and treatment with NAC + Vitamin A, in advanced cervical carcinoma. Both study groups received treatments consisting of a regime of cisplatin and paclitaxel. The study was conducted at the General Hospital of Dr. Mohammad Hoesin. The total number of patients recruited for the trial was 30 with 15 patients per treatment arm. One group received NAC consisting of cisplatin and paclitaxel and the remaining 15 patients received NAC + Vitamin A.

**Results:**

The addition of Vitamin A was found to be much better in influencing the clinical response in the treatment of advanced cervical carcinoma, although this was not statistically significant. However, a larger sample size with the reported proportion of higher positive outcome for NAC + Vitamin A may be statistically significant.

**Conclusion:**

Based on the results, Vitamin A supplementation in the treatment of advanced cervical carcinoma with neoadjuvant chemotherapy may play a crucial role in the treatment of cervical carcinoma.

## Introduction

According to the latest report from the World Health Organization (WHO), there are approximately 528,000 new cases of cervical carcinoma per year. Cervical cancer ranks fourth after breast, colorectal and lung cancer, and the incidence is highest in developing countries. Cervical cancer is also ranked as the fourth leading cause of death from cancer (266,000 deaths in 2012) in women worldwide, of which 70% occur in developing countries. In 2007, the number of cervical cancer patients reached 7.9 million and about 72% occurred in developing countries. Cancer deaths are projected to continue rising; numbers are expected to reach 12 million deaths in 2030 without adequate intervention (WHO, 2013; Ferlay et al., 2011).

High mortality rates of cervical carcinoma are mainly influenced by a late diagnosis in areas with limited resources, both in developed and developing countries (Smith et al., 2013). In Indonesia, the incidence of carcinoma in women is very high, with carcinoma of the cervix, uterus and breast ranked at the top. There is an estimated 180,000 new cancer cases annually, with the mortality rate expected to reach 75% within the first year of diagnosis. These deaths are associated with most stages of cervical cancer (70% of cases), including the invasive stage, continued/ recurring and even terminal stage at diagnosis (NCCN, 2014; Ferlay et al., 2011). 

Management of cervical carcinoma has many constraints due to the inadequate available facilities and infrastructure. This is, of course, very much influenced by the medical expense, especially at an advanced stage. A cancer survey in Japan found that most cancer patients have problems with the cost of treatment, and approximately 5.7% of patients drop out of treatment due to expensive treatment. The main difficulties cancer patients face is financial burden, physical pain, struggles with spiritual beliefs and emotional and social stress (Kamijo et al., 2014).

Primary chemo-radiation is the standard treatment offered to patients with advanced carcinoma (stage IIB-IVA). There are limitations to external and internal radiation facilities in Indonesia. This has led to a deluge of patients seeking treatment, thereby causing long waiting periods (3 to 12 months) for treatment. Patients on the waiting list are offered chemotherapy, or no treatment at all. Chemotherapy on its own is still questionable in terms of its effectiveness. The prolonged waiting period for surgery or radiation can have a detrimental effect on the health and prognosis of cancer patients, especially for those waiting for primary therapy with advanced stage carcinoma. In general, long waiting times are associated with poor service access or poor service quality (Yun et al., 2012).

Facilities and infrastructure needed in the treatment or management of carcinoma patients is complicated. Medication and means of therapies such as chemotherapy and radiotherapy are necessary to achieve optimal treatment. However, availability and access is still very limited, especially in developing countries. Specific to the present study, the only radiotherapy available at the Dr. Mohammad Hoesin Hospital in Indonesia is external radiation with Cobalt 60. Patients seeking internal radiation (brachytherapy) would be referred to a facility in Jakarta or given additional external radiation using box systems. This machine often is often broken or the source of radiation requires change. The latter unfortunately takes a long time to complete. Optimal treatment of early-stage carcinomas (stage IA1, IA2 and IB1) is dependent on the characteristic of the disease detected in each patient and consists of radical surgery or chemo-radiation. Surgery is not possible in advanced stage carcinomas (IIB-IVB). Chemo-radiation is the primary therapy that is performed in such cases (Lachance et al., 2008).

With their limited facilities, the choice of treatment of advanced cervical carcinoma in Dr. Mohammad Hoesin Hospital is influenced by many factors, such as the availability of radiotherapy, chemotherapy drug availability, and characteristics of the patient and the doctor responsible for the patient. The use of neoadjuvant chemotherapy (NAC) alone in advanced cervical cancer (late advanced cervical carcinoma = LACC) is still being debated and has not been included in the recommended oncology therapies by various international organizations. This study will contrast which method will provide a better prognosis compared with chemo-radiation therapy with stage IB2-IIB cervical cancer.

The rationale behind the use of NAC is to induce tumour shrinkage prior to pursuing radical excision and possibly improve the outcome compared to surgery alone. NAC also has the benefit of sterilizing lymph nodes and parametria, thereby lowering the risk of adjuvant therapy after surgery. However, the efficacy of the NAC in this condition is still unknown (Wiebea et al., 2012).

Cisplatin (DDP) is often used as a single chemotherapy regimen. Combination chemotherapy is set individually by an oncologist to consider the function of the heart, liver and kidneys. Wang et al., (2011) used BIP (DDP + bleomycin + ifosfamide) and the PF regimen (DDP + 5fluorouracil). They found NAC in patients with locally advanced cervical cancer had a beneficial effect on local control by improving the chance, and lowering the risks of surgery.

Yang et al., (2016) conducted a randomized multicentre study of 109 patients who underwent neoadjuvant chemotherapy (irinotecan and cisplatin or paclitaxel and cisplatin). The outcome of the treatment was compared directly with patients who received surgery (110 patients) in the treatment of locally advanced cervical cancer. The results of this study concluded that NAC did not improve overall survival but did reduce the number of patients who received post-operative radiotherapy (Yang et al., 2016).

In cervical cancers with the histopathological type of squamous cell carcinoma, cisplatin is the most active chemotherapeutic agent against cervical cancer with a response rate of 25%. Carboplatin has activity in cervical cancer with a response rate of 20%. Paclitaxel is known to have moderate activity in cervical cancer with a response rate of 17%. The success of the combination of paclitaxel-carboplatin is higher in squamous cell carcinoma due to each agent having high cytotoxic activity as a single agent (Hacker et al., 2001).

Malignant neoplasms derived from basal cells of the uterus, cervix and squamous cell carcinoma is very common and found in approximately 80-85% of cases. Chronic infection with human papilloma virus (HPV), in particular high-risk HPV16 and 18 variants, is an important etiologic factor in the pathogenesis of cervical carcinoma and its precursor. Multiple risk factors, such as sexual activity at a young age, multiple parity, smoking and use of oral contraceptives, are associated with cervical carcinoma. There is clear evidence that poor nutrition is also a risk factor for malignant transformation in cells of the cervix. Improving the nutritional status of patients plays a role in the prevention and repair of cervical dysplasia. Repair of cervical dysplasia, mild and moderate, have been shown in patients receiving Vitamin A supplements within a few months. A number of clinical studies have shown that topical application of Vitamin A in the cervix has shown full improvements of cervical dysplasia in 50% of cases (Osanai et al., 2014).

Vitamin A or retinol; with the active metabolite retinoic, has a role in the function of various cells. Retinol or retinoic acid controls cell proliferation through activities that stimulate the G1 rest phase and S rest phase. This mechanism occurs as retinol has a role in strengthening the expression of p53, p21 activity, and the activity of cyclin suppression (Whitworth et al., 2012; Budhu et al., 2002).

In addition, retinoic acid also plays a role in the strengthening of apoptosis in various cancer cells. Apoptosis is stimulated by retinoic acid going through enhancement of AP-1, stimulating caspase 7 and 9. Therefore, retinol/ retinoic acid have a suppressive effect on the growth of cancer cells or have hidden effects on the occurrence of cancer through two mechanisms: cell cycle and apoptosis (Budhu et al., 2002; Zhang et al., 2000).

Retinol enters cells via active mechanisms or through retinol receptors. Within the cell membrane, retinol is converted into retinoic acid. Retinoic acid signals apoptosis through the retinoic acid receptor complex. Retinoic acid will then enter the cell nucleus and bind to the retinoic acid receptors in the cell nucleus. The retinoic acid receptor complex will generate signals to the cell cycle rest phase at G1 and S, and apoptosis signal (Whitworth et al., 2012; Budhu et al., 2002; Zhang et al., 2000).

This study will conduct research related to the treatment of advanced cervical carcinoma with neoadjuvant chemotherapy existing standards, with the additional provision of Vitamin A (retinoid) during treatment at the Dr. Mohammad Hoesin Hospital to assess factors that could affect clinical treatment.

## Materials and Methods

This randomized double-blind clinical trial was conducted at the General Hospital of Dr. Mohammad Hoesin in Palembang, Indonesia, from January 2016 to September 2016, to compare the results of neoadjuvant chemotherapy (NAC) treatment with cisplatin and paclitaxel against neoadjuvant chemotherapy with added Vitamin A (NAC + Vitamin A) in advanced cervical carcinoma patients. The participant inclusion criteria included patients diagnosed with, and treated for, locally advanced cervical carcinoma. Patients with early stage cervical carcinoma; advanced cervical carcinoma who did not complete their treatment; advanced cervical carcinoma with chronic renal failure; advanced cervical carcinoma with systemic disease; and patients who died before completing treatment, were not included in this study.

A total of 30 patients were recruited, 15 of whom received neoadjuvant chemotherapy and 15 of whom received neoadjuvant chemotherapy with added Vitamin A. Vitamin A was administered in tablet form with a dose of 8.0000 IU per 8 hours. It was administered to participants from the start of their first cycle of chemotherapy to the start of their fourth cycle of chemotherapy (64 weeks). Primary endpoints were measurements of tumour mass before treatment and measurements of tumour mass before the fourth cycle of chemotherapy. The secondary endpoints were whether surgery could be attempted after chemotherapy and whether surgery could be attempted after chemotherapy with Vitamin A administration.

Patients were randomly assigned their groups. In general the characteristics of patients in both treatment groups were statistically similar. Mean age, mean parity and mean levels of serum Vitamin A were normally distributed, demonstrated by the independent T test. In addition, it has been demonstrated in this study that there were no differences in the type of histology, differentiation, lymphoma vascular involvement (LVSI) and the receptors of Vitamin A (p = 0.461) in both groups. The outcome of findings are summarized in Table 1.

*A. Data collection*

Participant information was collected from medical records; such as age, body weight, tumour histology and tumour diameter before treatment. Information regarding response to treatment, disease recurrence, vital status and complications of radiation were also collected.

Prior to treatment, patients underwent MRI examination to assess the size of the tumour, parametrial involvement, pelvic lymph node and para-aorta enlargement. Participants underwent MRI for therapeutic assessment after 3 cycles of chemotherapy in regard to changes in tumour mass, the parametria, and lymph nodes. 

*B. Processing and presentation of data*

Statistical analysis was conducted using SPSS. The significance of the continuous variables were tested using the student t-test (p= 0.05). The difference between the treatment groups were assessed using the Chi-square test. The collected data are presented in tables to observe the findings and conducted analyses with ease.

## Results

Comparison of the Effects of Intervention on Size of Cervical Tumours. Post intervention MRI examination demonstrated a significant reduction in the tumour volumes of patients in both treatment groups (NAC or group of NAC + Vitamin A). 

The volume of cervical tumours in patients with advanced cervical carcinoma was examined and measured using MRI. The findings on the sizes of tumours before and after treatment in both groups is summarized in Table 2. There were no statistical differences in the tumour size prior to either treatment (p = 0.885).

**Table 1:** Characteristics of Research Subjects

General characteristics	Group		*P*
	NAC +	NAC	
	Vitamin A		
Age (years)	54.0 ± 9.16	47.7 ± 8.216	0.058 *
Parity	4.33 ± 1915	3.67 ± 1,047	0.250 *
Levels of Serum Vitamin A (ug/L)	485.4 ± 172.89	417.71 ± 174.38	0.295 *
Histology type, n (%)			
squamous	11 (73.3)	8 (53.3)	0.450 **
Non Squamous	4 (26.7)	7 (46.7)	
Differentiation, n (%)			
Good-Moderate	14 (93.3)	12 (80)	0.598 **
Bad- Undifferentiated	1 (6.7)	3 (20)	
LVSI (lymphovascular involvement), n (%)			
Positive	9 (60)	9 (60)	1,000 **
Negative	6 (40)	6 (40)	
Receptors Vitamin A, n (%)			
Many + Medium	8 (53.3)	5 (33.3)	0.461 ***
Lightweight + No	7 (46.7)	10 (66.7)	

* , Independent T Test, *p *<0.05;

** , Fisher Exact, *p* <0.05;

*** , The Chi Square test, *p* <0.05

**Table 2:** Comparison of the Effects of Intervention on Size of Cervical Tumour

Variable	NAC +Vitamin A	P *	NAC	P *	P **
Tumour size					
Before	43,680 (19,155-78,980)	0.001	38,662 (13,698-110,443)	0.011	0.885
After	13,490 (8,744.7-30,784)		22,838 (86,96.7-98,800)		0.021

* Wilcoxon test, p <0.05;

** Mann Whitney test, p <0.05

**Table 3:** Characteristics of Clinical Response after Intervention

Characteristics	Group		P
	NAC +	NAC	
	vitamin A		
Clinical response, n (%)			
Response	11 (73.3 %)	6 (40 %)	0.139 *
No response	4 (26.7 %)	9 (60 %)	
Clinical response, n (%)			
Complete	3 (20)	1 (6.7)	0.164 **
Partial	8 (53.3)	5 (33.3)	
No Response	4 (26.7)	9 (60%)	

** , Fisher Exact test; p <0.05;

*** , The Chi Square test, p <0.05

**Table 4:** Comparison of Tumour Size Post-Treatment Based on Receptors of Vitamin A

General characteristics	Tumour Size Mass		*P*
	(NAC + Vitamin A)	NAC	
Receptors Vitamin A, n (%)			
High + Medium	14,712 (8,744-30,780)	24,864 (9,892--98,899)	0.834 *
Low + No	11,393 (9,415-24,530)	22,205 (8,696-48,140)	

The median and size range of the tumour prior to treatment in the NAC treatment group was 38,662 (range: 13,698-110,443) whereas the median and size range of the tumour after administration of NAC was 13,490 (range: 8,744.7 to 30,784). Statistical analyses of these findings revealed significant differences in the size of the tumour before and after administration of NAC (p = 0.011). In this treatment group the size of the tumour was reduced in size by 26.6% following treatment. 

The NAC + Vitamin A group had a median and tumour size range prior to treatment of 38662 (range: 13698-110443), reducing to 22838 (range: 8,696.7 – 98,800). Statistical analyses of these results revealed significant differences in the sizes of tumours before and after administration (p = 0.001). In this treatment group the reduction in sizes of the tumours averaged 65.5%. A statistical test of the difference between these two proportions indicated a significant difference [difference= 38.9%; 95%Cl: 3.44% to 63.22%; Chi-square: 4.416; DF: 1; Significance level: p=0.0356] between these two proportions, which indicates a significant difference between the results of the two treatments.

*Comparison of Clinical Response after Intervention*

The present study noted a clinical response of as much as 56.7% (n=17). However, as much as 43.3% (n=13) showed no clinical response, in particular the NAC group (60%). In contrast however, the group treated with NAC + Vitamin A demonstrated that a vast majority of the patients (73.3%) had a clinical response.

The recorded outcomes were complete response to treatment in 13.3% of patients, followed by partial response in 43.3% of the patients. The remaining 43.3% showed no response to treatment.

Observation of the group treated with NAC alone found that 6.7% of the patients obtained complete response, 33.3% partial response and in 60% no response. In contrast, the group given NAC + Vitamin A obtained a much higher response with 20% demonstrating a complete response, 53.3% partial response and 26.7% no response. It is readily obvious that the group given NAC + Vitamin A elicited a greater proportion of complete response to treatment than those administered NAC alone. This means the provision of Vitamin A influenced the clinical response to a greater degree in patients with advanced cervical carcinoma.

*Comparison of Tumour Size Post-Treatment Based on Receptors of Vitamin A*

The results of this study established that there are differences between the mean tumour size after treatment for both treatments, which exhibited receptors high + medium and receptors low + no (p = 0.834). It was concluded that Vitamin A beta receptors of advanced cervical tumour cells did not affect the occurrence of mass changes in tumours after treatment.

## Discussion

The significant reduction (p <0.05) observed in the volume of tumours post intervention in both treatments in the present study is a crucial finding because the size of the tumour and the tumour volume are recognized as an important prognostic factor for survival in patients suffering from cervical carcinoma. Careful clinical examination of MRI volumetric wear will provide an accurate picture of cervical and parametrium infiltration. NAC intervention can reduce tumours and lymph nodes, thereby minimizing the need for radiotherapy or chemoradiation. Decrease in tumour volume will make surgery; especially parametrium resection less invasive, thereby decreasing the number of complications that could occur. Decrease in tumour size and negative lymph node involvement will allow for less radical surgical procedures such as hysterectomies, radical hysterectomies or modified radical hysterectomies with nerve sparing.

There are several studies on the effectiveness of NAC treatment on reducing tumour mass in cases of advanced cervical carcinomas. Although the conventional treatment of cervical carcinoma is currently experiencing many challenges; several studies have confirmed NAC followed by radical surgery as an alternative, especially in countries where the radiation source is not available, and/or if patients still need to preserve their fertility despite the large tumour size. The International Atomic Energy Association (IAEA) reported that at least 30 developing countries, including 15 countries in Africa, do not have access to radiation therapy equipment (Schwab et al., 2014).

NAC is also reported to reduce the risk of pathological factors that may be associated with the prognosis for cervical cancer. Wang et al., (2011) cited the review of Guo et al., (2015) in which 110 patients with advanced stage cervical carcinoma underwent a radical hysterectomy with or without NAC. Sixty-eight patients were administered NAC with a base of platinum and then underwent surgery while the remaining 42 patients underwent surgery directly without NAC intervention. In this study 48 out of 68 (70.6%) patients who had prior NAC intervention had complete or partial remission compared to a much smaller response in patients subjected to operation without NAC treatment (Wang et al., 2011).

Ruiz and Hernandez conducted a search for relevant articles in EMBASE and PubMed-NCBI to identify potential interactions between the consumption or use of phytochemicals (carotenoids) and risk of cancer. Their search led to the conclusion that consumption of carotenoids (such as lycopene, alpha-carotene, and beta-carotene), reduced cancer risk, such as breast and prostate cancers (Ruiz and Hernandez, 2016). 

Retinoids and rexinoids are ligands which are recognized by specific receptors that directly interact with the genome and regulate concerted programs of gene expression. These programs elicit coordinated physiological changes that can antagonize malignant transformation, promote differentiation, cause apoptosis, insulin sensitivity, oxidative metabolism, and immune modulation, or suppress inflammation. These responses collectively exert antineoplastic effects. Retinoids as multifunctional agents are especially well-suited to combination therapy (Uray et al., 2016).

In this study, MRI performed before and after NAC administration noted the tumour diameter was reduced by as much as 2.57 ± 1.90 cm (P <0.01). Based on the results of the present investigation, the author feels that further investigations to determine the benefits of Vitamin A supplementation during NAC in the reduction of tumour mass has merit.

The comparatively greater tumour size reduction noted in Vitamin A + NAC group compared to the group NAC alone (p = 0.021) suggests that the supplementation of Vitamin A influences and mediates changes to the tumour volume of cervical carcinomas.

Studies have shown that there is a role for antioxidants in decreasing the incidence of cervical carcinoma. In a case-controlled study on Chinese women, Guo et al., (2015) assessed the effect of diet and serum vitamin antioxidants on 458 women with invasive cervical carcinoma and 742 healthy controls. They noted that there was a correlation between high serum vitamin antioxidants and low incidence of cervical carcinoma with odds ratios (ORs) of 0.66 (95% confidence interval [CI] = 0.46-0.93; p = 0.024) for α-carotene, 0.63 (95% CI = 0.45-0.90; p = 0.006) for β-carotene, 0.53 (95% CI = 0.37-0.74; p <0.001) for Vitamin E, 0.48 (95% CI = 0.33-0.69; p <0.001). Vitamin C also had a similar impact. The authors concluded that their findings are indicative that antioxidants and vitamins (especially α-carotene, β-carotene, and Vitamins E and C) have a beneficial effect in reducing the incidence of invasive cervical carcinoma (Guo et al., 2015).

In their report, Martos et al., (2014) examined the levels of Vitamin A (retinol and α-carotene) in breast carcinoma before and after radiation in 230 patients (mean age 63.6 years ± 9.38). They observed that decrease in serum retinol was significant at 45.1 ± 18.2 mg/dL before treatment, compared to 27.1 ± 11.7 mg/dL after treatment (p <0.001). Likewise, β-carotene level was 209.0 ± 153.6 pg/L compared to 47.7 ± 25.5 pg/L (p <0.001). Significant differences in serum retinol (p<0.001) and β-carotene (p=0.003,) were also observed based on the stage of the disease. They recommended the provision of appropriate nutrition to overcome deficiencies, in particular of antioxidants, to minimize the harmful effects of radiation (Martos et al., 2014).

In the present investigation, both patient groups had similar (p = 0.295) levels of Vitamin A prior to treatment. Following treatment with NAC and NAC + Vitamin A, the response outcome of the treatment was significantly more effective with Vitamin A supplementation, which is indicative of the beneficial effects of Vitamin A supplementation during NAC administration. The results of the present study points toward a role for Vitamin A in the treatment of cervical carcinoma as it appears to be involved in reducing size of tumours. The present findings are supported by numerous other studies, even though there appears to be little correlation and consistency between dietary factors and risk of cervical carcinoma in large-scale studies, observational research as well as randomized clinical trials. In this study, radiological response was investigated after 3 cycles of chemotherapy to assess changes in tumour size to aid with the decision to continue treatment with surgery. The results demonstrated differences in tumour size. Further evaluation was made to investigate whether there is a difference in therapeutic response. A difference was not found, indicating that further assessment of therapy is needed after 6 cycles of chemotherapy. 

In conclusion, although tumour shrinkage in patients administered NAC + Vitamin A does not appear to be significantly different (p=0.139) to the shrinkage observed with NAC alone, it is apparent that the shrinkage was more pronounced (75%) when Vitamin A was administered together with NAC as opposed to the lower shrinkage (40%) observed with NAC alone. This is indication that Vitamin A may have enhanced the treatment outcome observed in the present study. It appears highly plausible that if the sample size was larger, the difference between percentage tumour shrinkage for the treatments groups of NAC and NAC + Vitamin A may be more statistically significant.

The results of this study indicate that the addition of Vitamin A caused a statistically significant reduction in tumour size of cervical carcinomas. In the event a statistically insignificant positive response is observed after three cycles of chemotherapy, it appears justified to continue with chemotherapy. In the light of past and present findings, it is reasonable to speculate that the significant reduction in tumour volume may well be attributed to the combined effects of NAC and Vitamin A. It therefore appears reasonable to suggest that Vitamin A supplementation should be provided on a routine basis in the treatment of advanced cervical carcinoma with neoadjuvant chemotherapy (NAC). 
